# One-year follow-up effects of an acceptance-based treatment for hypersexuality

**DOI:** 10.3389/fpsyg.2026.1706722

**Published:** 2026-01-29

**Authors:** Marta Ortega-Otero, Eduardo Polín, David Lobato, Francisco Montesinos

**Affiliations:** Department of Psychology, Faculty of Biomedical and Health Sciences, Universidad Europea de Madrid, Madrid, Spain

**Keywords:** Acceptance and Commitment Therapy, ACT, chemsex, hypersexuality, LGBTQIA+ mental health, psychological flexibility, sexual health

## Abstract

**Background:**

Hypersexuality is associated with significant psychological distress and health risks, yet few studies have examined the long-term effects of psychological interventions.

**Objective:**

This study aimed to evaluate the one-year follow-up outcomes of a brief, individual Acceptance and Commitment Therapy (ACT) intervention for patients with clinically significant hypersexuality.

**Methods:**

Ten participants who completed an eight-session ACT protocol were assessed at pre-treatment, post-treatment, and at 3-month and 1-year follow-ups using standardized self-report measures. Statistical and clinical significance were analyzed using repeated measures ANOVA, Friedman tests, and the Jacobson and Truax method to evaluate individual-level change.

**Results:**

Reductions in hypersexuality and craving interference, as well as increases in values-consistent behavior and perceived control over craving, were maintained at the one-year follow-up. Psychological inflexibility, which had not significantly changed immediately after treatment, showed significant improvement at 1-year follow-up. Clinically significant reductions in hypersexuality were observed in 9 out of 10 participants, while changes in psychological inflexibility were more limited.

**Conclusion:**

These findings provide preliminary evidence for the sustained effectiveness, feasibility, and acceptability of a brief ACT-based intervention for hypersexuality, particularly among men with non-heterosexual orientations and individuals engaging in chemsex. Further controlled trials are warranted to confirm these results.

## 1 Introduction

It is estimated that between 3% and 6% of individuals experience hypersexuality (Castro-Calvo et al., 2017; Derbyshire and Grant, 2015; Karila et al., 2014; Kingston and Firestone, 2008). Hypersexuality has been defined as the uninhibited or excessive engagement in culturally normative, non-paraphilic sexual behaviors, characterized by a high frequency and intensity of sexual fantasies, arousal, or impulsive sexual actions (Kafka, 2010), such as masturbation—with or without pornography use—or sexual intercourse. There is ongoing debate regarding what frequency of sexual activity may be considered excessive (Kaplan and Krueger, 2010), and no consensus has been reached on the most appropriate term to describe this phenomenon. In this context, hypersexuality is not defined merely by behavioral parameters—nor by frequency alone—but rather by the extent to which these behaviors negatively affect an individual’s overall functioning (Perrotta, 2023). There is substantial evidence that this uninhibited sexual expression can lead to significant adverse consequences, including personal distress, sexually transmitted infections, unplanned pregnancies, relationship difficulties, and financial, occupational, and academic problems (Kaplan and Krueger, 2010).

Two distinct subcomponents of hypersexuality have been identified: “problematic sexuality,” which refers to sexual compulsivity and the use of sex as a coping mechanism, and “sexual drive,” which includes preoccupation with sexual fantasies and heightened sexual arousal (Du and Knight, 2024). Alternative terms have been proposed, such as “compulsive sexual behavior” (Kuzma and Black, 2008) and “sexual addiction” (Carnes, 1990). Although the inclusion of hypersexuality as a distinct disorder was not accepted in the Diagnostic and Statistical Manual of Mental Disorders, Fifth Edition (DSM-5; American Psychiatric Association, 2013), some scholars argue that hypersexuality may represent a manifestation of other underlying psychopathological conditions. In this regard, factors that may concur with—or contribute to—its development include mood and bipolar disorders, obsessive-compulsive disorder, emotional dysregulation, and trauma (Limoncin et al., 2022). However, in the International Classification of Diseases (ICD-11; World Health Organization, 2019), it has been classified as “compulsive sexual behavior disorder” under the category of impulse control disorders.

Hypersexuality has been found to be more prevalent among males (Ballester-Arnal et al., 2013; Castro-Calvo et al., 2017; Dodge et al., 2004; Odlaug et al., 2013; Origlia et al., 2025; Skegg et al., 2010), and particularly among gay men (Daneback et al., 2005). Moreover, it has been associated with depression and substance use disorders (Ballester-Arnal et al., 2020), as well as anxiety (Bancroft and Vukadinovic, 2004), emotion dysregulation and impulsivity (Hegbe et al., 2021) and personality disorders (Kalichman and Rompa, 2001).

A strong association has also been identified between hypersexuality and difficulties in emotion regulation and coping with negative mood states (Lew-Starowicz et al., 2020; Miner et al., 2019). Additionally, hypersexuality has been linked to psychological inflexibility (Ortega-Otero et al., 2023), suggesting that compulsive sexual behaviors may operate as avoidance strategies aimed not only at reducing sexual urges but also at alleviating distressing emotional states. Among chemsex users, psychological inflexibility showed the strongest association with both hypersexuality and substance use compared with other variables (Rico and Montesinos, 2025). Experiential avoidance has also been found to be significantly associated with hypersexual behavior (Fadaei et al., 2025) and has been identified as a factor that partially explains the relationship between symptoms of anxiety and depression and compulsive sexual behavior. Accordingly, experiential avoidance has been proposed as a key maintenance mechanism of compulsive sexual behavior, consistent with models of maladaptive emotion regulation (Brem et al., 2017). This suggests that sexual behavior may function as a strategy of experiential avoidance over time.

Among the psychological treatments proposed for addressing hypersexuality, cognitive-behavioral therapies have demonstrated benefits such as increased psychological well-being (Hallberg et al., 2019; Hallberg et al., 2020), reduced depressive symptoms (FirooziKhojastehfar et al., 2021), and decreased time spent engaging in sexual behaviors (García-Barba, 2022). Similarly, Acceptance and Commitment Therapy (ACT), a third-wave cognitive-behavioral approach (Hayes et al., 2011), has shown promising results in reducing the frequency of sexual behaviors and alleviating related worries (Crosby and Twohig, 2016; FirooziKhojastehfar et al., 2021; Levin et al., 2017; Twohig and Crosby, 2010). However, existing clinical trials using ACT have primarily focused on problematic pornography use.

Nevertheless, promising ACT-informed studies have recently been published in areas related to hypersexuality other than problematic pornography use. For example, Strika-Bruneau et al. (2024a) demonstrated the feasibility of an approach designed to simultaneously treat an adult male’s sexual and cannabis addictions, along with symptoms of depression and anxiety. Likewise, emerging studies are beginning to explore the potential utility of ACT in individuals engaging in problematic chemsex or sexualized drug use. For instance, in the study conducted by Strika-Bruneau et al. (2024b), ACT was delivered in 13 to 16 individual sessions to 10 men who have sex with men (MSM) practicing chemsex, resulting in significant improvements in psychological flexibility, anxiety, depression, and the intensity of sexual addiction. Additionally, a case study published by our research team examined the effects of a brief ACT-based intervention in an HIV-positive MSM with hypersexuality and chemsex behaviors (Montesinos and Ortega, 2022). The intervention was followed by a reduction in hypersexual behaviors, specifically in the time spent seeking sexual encounters, the number of sexual partners, the frequency of sexual activity, and the use of substances during sexual encounters.

Subsequently, our team conducted an open-label pilot study involving 12 participants (83.3% male; 66.7% MSM; 66.7% engaged in chemsex) who presented clinically significant levels of hypersexuality (Montesinos et al., 2024). All participants received the same ACT-based protocol across eight weekly individual sessions, aimed at enhancing psychological flexibility. The treatment was completed by all participants, who reported high levels of satisfaction. The intervention resulted in statistically significant reductions in hypersexuality, which were maintained at a three-month follow-up. Large effect sizes were observed for hypersexuality, cognitive fusion, psychological inflexibility, mindfulness skills, body awareness, and sexual satisfaction. Post-treatment and follow-up assessments revealed significant improvements in hypersexuality, psychological flexibility, cognitive fusion, and mindfulness, all with large effect sizes. Furthermore, the analysis of clinical significance indicated that the majority of participants experienced clinically meaningful reductions in hypersexuality, accompanied by behavioral changes consistent with those observed in the previous case study.

More research is needed to determine whether individuals with hypersexuality who undergo ACT-based treatment experience relapses and return to previous levels of hypersexuality, or whether they continue to improve over time and experience long-term benefits following the intervention. This article extends the previous pilot study by aiming to analyze the long-term evolution of these participants after completing the eight-session individual ACT intervention. It reports the 1-year follow-up outcomes from a previously conducted pilot intervention, rather than presenting a cross-sectional analysis. Specifically, it seeks to determine whether the improvements observed post-intervention and at the three-month follow-up are sustained 1 year after treatment. The present study is explicitly framed within contemporary psychological science, grounding its hypotheses, methods, and interpretation of findings in empirically supported models of behavioral regulation, experiential avoidance, and psychological flexibility. The hypothesis is that the reduction in hypersexuality would be maintained at the one-year follow-up, along with improvements in cognitive fusion, psychological inflexibility, mindfulness skills, body awareness, and sexual satisfaction.

## 2 Method

### 2.1 Participants

Of the 12 individuals with clinically significant levels of hypersexuality (defined as a score greater than 53 on the Hypersexual Behavior Inventory) who completed the intervention and the three-month follow-up, 10 participants (83.3%) provided data at the one-year follow-up. Two participants did not respond to the investigators’ request for follow-up information. Among the 10 respondents, 80% were male, 70% identified as gay or bisexual. The mean age was 37.2 years (SD = 11.41). Seventy percent of the participants were originally from Spain, and the same proportion resided in Madrid. Most participants (70%) had completed university-level education, 70% were employed, and the majority (80%) were single.

### 2.2 Design

The study was designed as an open-label pilot trial with a single-center, comparative design and no control group. Standardized self-report measures were administered at four time points: pre-treatment, post-treatment, and at three- and 1-year follow-ups. Full methodological details, including inclusion and exclusion criteria, are provided in the initial report of the parent study.

### 2.3 Measures

#### 2.3.1 Primary variable

##### 2.3.1.1 Hypersexuality

To assess the degree of hypersexuality, the Spanish version of the Hypersexual Behavior Inventory (HBI; Reid et al., 2011), validated by Ballester-Arnal et al. (2019), was used. This instrument comprises 19 items rated on a 5-point Likert scale ranging from “never” to “often.” Total scores range from 19 to 95, with scores equal to or greater than 53 indicating clinically significant hypersexuality (Reid et al., 2011). The Spanish adaptation of the HBI has demonstrated high reliability, with Cronbach’s alpha values ranging from 0.89 to 0.96 (Ballester-Arnal et al., 2019).

#### 2.3.2 Secondary variables

##### 2.3.2.1 Psychological inflexibility

To assess psychological inflexibility, the Spanish version of the Acceptance and Action Questionnaire-II (AAQ-II; Bond et al., 2011), adapted by Ruiz et al. (2013), was administered. This instrument consists of 7 items rated on a 7-point Likert scale ranging from “never true” to “always true.” Higher scores reflect greater psychological inflexibility. The Spanish version has demonstrated good internal consistency (*α* = 0.74), comparable to that of the original version (Barraca, 2004), and has shown adequate construct, discriminant, and external validity (Ruiz et al., 2013).

##### 2.3.2.2 Sexual satisfaction

The Spanish adaptation of the New Sexual Satisfaction Scale–Short Form (NSSS-S; Štulhofer et al., 2010) by Strizzi et al. (2016) was employed to assess sexual satisfaction. This instrument comprises 12 items rated on a 5-point Likert scale ranging from “not at all satisfied” to “extremely satisfied.” It has been shown to possess adequate internal consistency, with a Cronbach’s alpha of 0.92 (Strizzi et al., 2016).

##### 2.3.2.3 Body connection

The Scale of Body Connection (SBC; Price and Thompson, 2007), Spanish version by Quezada-Berumen et al. (2014), was used to assess body awareness and bodily dissociation. The instrument includes 20 items rated on a 5-point Likert scale ranging from “not at all or never” to “all the time.” The Body Awareness (BA) subscale evaluates attentiveness to internal bodily experiences, while the Bodily Dissociation (BD) subscale measures the tendency to avoid such experiences. The Spanish version demonstrated acceptable internal consistency, with Cronbach’s alpha values of 0.86 for BA and 0.62 for BD (Quezada-Berumen et al., 2014).

##### 2.3.2.4 Cognitive fusion

To assess cognitive fusion, the Spanish version of the Cognitive Fusion Questionnaire (CFQ; Gillanders et al., 2014), adapted by Romero-Moreno et al. (2014), was used. This instrument consists of 7 items rated on a 7-point Likert scale ranging from “never true” to “always true.” Higher scores indicate a stronger tendency to accept the literal content of internal experiences, reflecting greater cognitive fusion—defined as the dominance of verbal processes over behavioral regulation, to the exclusion of other sources of stimulus control (Hayes et al., 2014). The Spanish version demonstrated good internal consistency, with a Cronbach’s alpha of 0.87 (Romero-Moreno et al., 2014).

##### 2.3.2.5 Mindfulness

The Spanish version of the Mindful Attention Awareness Scale (MAAS; Brown and Ryan, 2003), adapted by Soler et al. (2012), was used to assess mindfulness-related skills. The scale consists of 15 items rated on a 6-point Likert scale ranging from “almost always” to “almost never,” with higher scores indicating greater attention to present-moment experiences. The Spanish adaptation demonstrated high internal consistency (*α* = 0.89), comparable to that of the original version (Soler et al., 2012).

##### 2.3.2.6 Values

The Valued Living Questionnaire (VLQ; Wilson et al., 2010) was used to evaluate individuals’ personal values across various life domains. The questionnaire comprises three core dimensions: importance, consistency, and discrepancy. Participants rate the importance of each domain (e.g., family, relationships, work, health, spirituality) on a Likert scale ranging from 1 (not at all important) to 10 (extremely important). Consistency is assessed by asking participants to rate how consistently they have acted in accordance with their values in each domain over the past week, using the same 10-point scale (1 = completely inconsistent, 10 = completely consistent). Discrepancy is calculated as the absolute difference between the importance and consistency scores for each domain, reflecting the degree of misalignment between values and behavior. Psychometric properties of the Spanish adaptation of the questionnaire (Macías et al., 2023) indicate acceptable internal consistency, with Cronbach’s alpha coefficients between 0.68 and 0.71.

##### 2.3.2.7 Interference

The degree of craving-related interference was assessed using an ad-hoc, subjective Likert-type scale ranging from 1 (very low interference) to 10 (very high interference). To help participants contextualize their responses, illustrative examples were provided, such as: “It significantly distances me from the life I would like to have, and from the things that matter most to me, such as my job, my health, or my romantic relationship.”

##### 2.3.2.8 Control

Perceived control over craving was measured using an ad-hoc, subjective Likert-type scale ranging from 1 (no control at all) to 10 (complete control).

### 2.4 Procedure

The study received ethical approval from the Ethics Committee of Universidad Europea (approval code: CIPI/22.248) and was also registered as an IRB-approved clinical trial (ClinicalTrials.gov Identifier: NCT05572372). Participation was voluntary, and individuals who met the inclusion criteria were asked to sign an informed consent form, which guaranteed the confidentiality of their data. Recruitment for the open-label trial took place between January and May 2022 through advertisements on social media and outreach via non-profit organizations serving the LGBTQIA+ and HIV communities. Participants were recontacted via email for the 1-year follow-up between May and August 2023. The intervention was delivered by the first and last authors and an external collaborator, all trained in contextual therapies. The last author and the collaborator were experienced ACT practitioners with postgraduate training, while the first author had foundational ACT training and worked under the first author’s supervision. Data collection was conducted using standardized self-report questionnaires administered through Google Forms.

### 2.5 Intervention

Details regarding the therapeutic methods and their alignment with ACT core processes and session content are available in Montesinos et al. (2024) and a detailed version of the protocol was previously published in a case study (Montesinos and Ortega, 2022). The intervention consisted of eight individualized online sessions, conducted weekly and lasting approximately 1 hour each. The protocol was tailored to each participant based on a functional analysis and incorporated ACT-consistent techniques such as metaphors and experiential exercises designed to promote the six core processes of psychological flexibility: acceptance, cognitive defusion, present-moment awareness, values clarification, self-as-context, and committed action.

For values clarification, exercises such as the “funeral exercise” (Hayes et al., 2014, p. 446) and the “garden metaphor” (Hayes et al., 2014, p. 483) were used. To illustrate the challenges of controlling private experiences, the “hungry tiger” metaphor (Eifert and Forsyth, 2005) and the “chocolate cake” task (Hayes et al., 2014, p. 278) were employed. Techniques aimed at fostering acceptance, cognitive defusion, awareness of internal experiences, and recognition of avoidance consequences—as well as promoting the self-as-context perspective—included body scan meditation (Kabat-Zinn, 2013), the physicalizing exercise (Hayes et al., 2014, p. 417), placing triggering thoughts on signs (adapted from the “marching soldiers” exercise, Hayes et al., 2014, p. 374), the “shopping street” metaphor (adapted from Wilson and Luciano, 2002, p. 115), and a variation of the “autumn and leaves” exercise (Wilson and Luciano, 2002, p. 216). For relapse prevention, the “falling off the bike” metaphor (adapted from the rider metaphor, Hayes et al., 2014) was utilized.

### 2.6 Statistical analyses

First, means and standard deviations were calculated for all dependent variables at four time points: pre-treatment, post-treatment, and at three- and 1-year follow-ups. The Shapiro–Wilk test was used to assess the normality of the data across these time points. Those variables that followed a normal distribution in all the time points and did not violate the assumption of sphericity were analyzed through parametric tests.

Second, two complementary strategies were employed to evaluate the effectiveness of the intervention at the 1-year follow-up. On one hand, repeated measures ANOVA was conducted, with general effect sizes reported using partial eta squared (η^2^ₚ), and *post hoc* comparisons performed using paired *t*-tests (effect sizes for the post-hoc comparisons were reported through Cohen’s d). The assumption of sphericity was tested with Mauchly’s test. When this assumption was violated, the non-parametric Friedman test was used instead of ANOVA to determine statistical significance. In such cases, post hoc comparisons were conducted using the Conover test, and effect sizes were calculated using Kendall’s W (for general analyses) and Rank-Biserial correlations (for post-hoc analyses).

These analyses were carried out with the software JASP, version 0.18.3.

On the other hand, clinical significance was assessed using the method proposed by Jacobson and Truax (1991). For each outcome measure, the Reliable Change Index (RCI) was calculated in order to determine whether the magnitude of change observed at the individual level exceeded what could be expected due to measurement error alone. A 95% confidence interval was applied, such that absolute RCI values greater than ±1.96 were interpreted as indicating statistically reliable change. In addition, clinical significance was evaluated using Jacobson and Truax’s Cutoff A criterion, defined as a score located at least two standard deviations beyond the pretreatment mean, indicating movement toward a functional range. This criterion was selected because the study involved a homogeneous clinical sample and external normative data from comparable functional populations were not available for all outcome measures. Pretreatment standard deviations of the sample and reliability estimates derived from Cronbach’s alpha coefficients reported in Spanish validation studies or from the present sample were used for these calculations.

## 3 Results

### 3.1 Parametric within-group results

**Figure 1:**
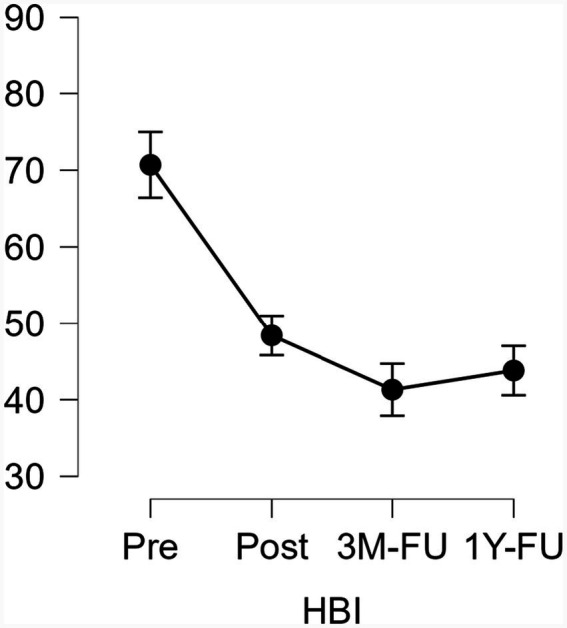
Mean HBI scores across time points. 3M-FU, Three-month follow-up; 1Y-FU, One-year follow-up; HBI, Hypersexual Behavior Inventory. Vertical bars represent the SEM (standard error of the mean).

A repeated measures ANOVA was conducted for each variable that did not violate the assumption of sphericity. Statistically significant differences with large effect sizes were found for hypersexuality (HBI) [*F*(3, 27) = 15.330, *p* < 0.001, η^2^ₚ = 0.630], psychological inflexibility (AAQ-II) [F(3, 27) = 3.153, *p* = 0.041, η^2^ₚ = 0.259], and importance (VLQ) [F(3, 27) = 3.882, *p* = 0.020, η^2^ₚ = 0.301]. Regarding hypersexuality, significant differences were observed between pre-treatment and post-treatment, pre-treatment and 3-month follow-up, and pre-treatment and 1-year follow-up (see Figure 1). No significant differences were found between post-treatment and 1-year follow-up, nor between the 3-month and 1-year follow-ups. These findings suggest that the reductions in hypersexuality achieved after the intervention were maintained 1 year later.

The means and standard deviations of all the studied variables, as well as the results of Shapiro–Wilk tests, are shown in Table 1. In terms of psychological inflexibility, significant reductions were observed between pre-treatment and the 1-year follow-up. However, no significant differences were found between pre- and post-treatment, nor between pre-treatment and the 3-month follow-up. Interestingly, the differences that were not significant at post-treatment or at the 3-month follow-up became significant at the 1-year follow-up. Regarding importance, significant changes were found only between pre- and post-treatment. These post-hoc analyses are detailed in Table 2. Overall, these patterns indicate that the intervention was followed by meaningful and durable improvements in key clinical processes, with some effects emerging or consolidating over time.

**Table 1:** Descriptive statistics and Shapiro–Wilk test for each measurement.

Measurement	Mean	SD	Shapiro–Wilk	*p*-value of Shapiro–Wilk
HBI Pre	70.70	15.17	0.90	0.237
HBI Post	48.40	14.27	0.99	0.998
HBI 3M-FU	41.30	21.31	0.88	0.130
HBI 1Y-FU	43.80	20.87	0.91	0.326
NSSS Pre	36.00	7.97	0.94	0.555
NSSS Post	36.90	6.67	0.92	0.416
NSSS 3M-FU	37.10	11.49	0.95	0.714
NSSS 1Y-FU	41.70	10.28	0.97	0.932
AAQ Pre	30.00	9.95	0.84	0.053
AAQ Post	26.00	9.35	0.88	0.158
AAQ 3M-FU	26.10	10.46	0.93	0.487
AAQ 1Y-FU	23.00	9.17	0.87	0.128
CFQ Pre	29.60	9.84	0.92	0.366
CFQ Post	24.10	9.37	0.87	0.125
CFQ 3M-FU	25.80	9.46	0.98	0.985
CFQ 1Y-FU	23.60	10.11	0.93	0.485
SBC BA Pre	28.60	8.98	0.91	0.321
SBC BA Post	30.00	5.09	0.90	0.237
SBC BA 3M-FU	31.90	7.85	0.95	0.670
SBC BA 1Y-FU	32.10	7.72	0.96	0.875
SBC BD Pre	13.90	6.79	0.85	0.059
SBC BD Post	11.10	5.54	0.89	0.198
SBC BD 3M-FU	12.40	6.39	0.94	0.588
SBC BD 1Y-FU	10.60	5.56	0.95	0.682
MAAS Pre	3.16	0.96	0.91	0.330
MAAS Post	3.40	0.97	0.93	0.468
MAAS 3M-FU	3.70	0.82	0.78	0.008*
MAAS 1Y-FU	3.80	1.19	0.91	0.327
IMP Pre	63.40	11.82	0.86	0.095
IMP Post	74.10	12.06	0.87	0.102
IMP 3M-FU	69.80	14.68	0.95	0.676
IMP 1Y-FU	69.00	18.08	0.96	0.873
CONS Pre	50.30	14.02	0.85	0.068
CONS Post	65.80	15.52	0.82	0.030*
CONS 3M-FU	60.00	13.94	0.87	0.103
CONS 1Y-FU	62.00	20.18	0.97	0.913
DISCR Pre	13.10	14.07	0.87	0.104
DISCR Post	7.50	10.16	0.92	0.413
DISCR 3M-FU	9.80	11.15	0.98	0.963
DISCR 1Y-FU	7.00	8.91	0.96	0.843
Interference Pre	6.60	2.06	0.93	0.447
Interference Post	3.40	2.45	0.95	0.749
Interference 3M-FU	2.50	2.01	0.93	0.537
Interference 1Y-FU	3.50	1.65	0.92	0.422
Control Pre	1.70	1.25	0.92	0.436
Control Post	4.00	2.16	0.92	0.393
Control 3M-FU	2.30	1.56	0.84	0.047*
Control 1Y-FU	6.30	3.19	0.84	0.054

SD, standard deviation; 3M-FU, three-month follow-up; 1Y-FU, one-year follow-up; HBI, Hypersexual Behavior Inventory; NSSS, The New Sexual Satisfaction Scale-short form; AAQ, Acceptance and Action Questionnaire II; CFQ, Cognitive Fusion Questionnaire; SBC BA, Scale of Body Connection, body awareness; SBC BD, Scale of Body Connection, bodily dissociation; MAAS, Mindful Attention Awareness Scale; IMP, importance; CONS, consistency; DISCR, discrepancy.

**Table 2:** *Post-hoc* comparisons between measurements of hypersexuality, psychological inflexibility and importance.

Measurements		Mean difference	SE	*t*	Cohen’s *d*	p_holm_
HBI Pre	HBI Post	22.30	4.84	4.59	1.22	<0.001***
	HBI 3M-FU	29.40	4.84	6.06	1.61	<0.001***
	HBI 1Y-FU	26.90	4.84	5.54	1.47	<0.001***
HBI Post	HBI 3M-FU	7.10	4.84	1.46	0.39	0.464
	HBI 1Y-FU	4.60	4.84	0.94	0.25	0.703
HBI 3M-FU	HBI 1Y-FU	−2.50	4.84	−0.51	−0.13	0.703
AAQ Pre	AAQ Post	4.00	2.28	1.75	0.41	0.457
	AAQ 3M-FU	3.90	2.28	1.7	0.4	0.457
	AAQ 1Y-FU	7.00	2.28	3.06	0.71	0.030*
AAQ Post	AAQ 3M-FU	−0.10	2.28	−0.044	−0.01	0.965
	AAQ 1Y-FU	3.00	2.28	1.31	0.30	0.559
AAQ 3M-FU	AAQ 1Y-FU	3.10	2.28	1.35	0.31	0.559
IMP Pre	IMP Post	−10.70	3.15	−3.39	−0.74	0.013*
	IMP 3M-FU	−6.40	3.15	−2.02	−0.44	0.263
	IMP 1Y-FU	−5.60	3.15	−1.77	−0.38	0.349
IMP Post	IMP 3M-FU	4.30	3.15	1.36	0.29	0.369
	IMP 1Y-FU	5.10	3.15	1.61	0.35	0.353
IMP 3M-FU	IMP 1Y-FU	0.80	3.15	0.25	0.05	0.802

Holm correction was applied. *p < 0.05; ***p < 0.001; 3M-FU, three-month follow-up; 1Y-FU, one-year follow-up; SE, standard error; HBI, hypersexual behavior inventory; AAQ, acceptance and action questionnaire II; IMP, importance. Cohen’s *d* interpretation (in absolute values): 0.2–0.49 small effect size. 0.5–0.79 medium effect size. ≥0.8 large effect size.

### 3.2 Non-parametric within-group results

The Friedman test was conducted for each variable that violated the assumption of sphericity. Statistically significant differences with moderate to large effect sizes were found across the different time points for consistency [χ^2^(3) = 10.316, *p* = 0.016, W = 0.344], interference [*χ*^2^(3) = 17.159, *p* < 0.001, W = 0.572], and control [χ^2^(3) = 14.596, *p* = 0.002, W = 0.487].

*Post-hoc* comparisons revealed significant differences in the variable interference between pre-treatment and 3-month follow-up. No significant differences were observed between pre- and post-treatment, post-treatment and 1-year follow-up, or between the two follow-ups. These results indicate that reductions in craving interference were sustained 1 year after the intervention. Regarding control over craving, significant differences were found between pre-treatment and 1-year follow-up. No significant differences were observed between post-treatment and 1-year follow-up, suggesting that the increase in perceived control over craving achieved after the intervention was also maintained 1 year later. Table 3 presents these post-hoc analyses. The remaining variables were analyzed using the same procedure, but no statistically significant differences were found. Taken together, these patterns indicate that the intervention was followed by consistent and enduring improvements across craving-related processes, with gains largely maintained at long-term follow-up.

**Table 3:** Conover’s post hoc comparisons between measurements of consistency, interference and control.

Measurements		T-Stat	df	W_i_	W_j_	r_rb_	p_holm_
CONS Pre	CONS Post	2.76	27	17.00	33.00	−0.891	0.061
	CONS 3M-FU	0.60	27	17.00	20.50	−0.709	1.000
	CONS 1Y-FU	2.15	27	17.00	29.50	−0.600	0.199
CONS Post	CONS 3M-FU	2.15	27	33.00	20.50	1.000	0.199
	CONS 1Y-FU	0.60	27	33.00	29.50	0.133	1.000
CONS 3M-FU	CONS 1Y-FU	1.55	27	20.50	29.50	−0.491	0.395
Int Pre	Int Post	1.90	27	36.00	26.00	0.844	0.203
	Int 3M-FU	4.09	27	36.00	14.50	1.000	0.002**
	Int 1Y-FU	2.38	27	36.00	23.50	1.000	0.123
Int Post	Int 3M-FU	2.18	27	26.00	14.50	0.667	0.150
	Int 1Y-FU	0.47	27	26.00	23.50	−0.133	0.638
Int 3M-FU	Int 1Y-FU	1.71	27	14.50	23.50	−1.000	0.203
Control Pre	Control Post	2.45	27	16.00	29.50	−0.844	0.083
	Control 3M-FU	0.72	27	16.00	20.00	−0.333	0.742
	Control 1Y-FU	3.36	27	16.00	34.50	−1.000	0.014*
Control Post	Control 3M-FU	1.72	27	29.50	20.00	1.000	0.286
	Control 1Y-FU	0.91	27	29.50	34.50	0.473	0.742
Control 3M-FU	Control 1Y-FU	2.63	27	20.00	34.50	0.889	0.068

Holm correction was applied. *p < 0.05; **p < 0.01; 3M-FU, three-month follow-up; 1Y-FU, one-year follow-up; df, degrees of freedom; CONS, consistency; Int, Interference; rrb, rank-biserial correlation. Rank-biserial correlation interpretation (in absolute values): 0.1–0.24 small effect size. 0.25–0.36 medium effect size. ≥0.37 large effect size.

### 3.3 Results of the intrasubject analysis

Clinical significance was assessed using the Jacobson and Truax (1991) method. A clinically significant reduction in hypersexuality (HBI) was observed in all participants following the intervention. This improvement was maintained in 9 out of 10 participants at both the 3-month and 1-year follow-ups. In contrast, no clinically significant changes in psychological inflexibility (AAQ-II) were observed immediately after the intervention. However, at the 3-month follow-up, one participant demonstrated a clinically significant reduction in psychological inflexibility, and by the 1-year follow-up, two participants had achieved this improvement. These results are summarized in Table 4. Overall, these findings show that while clinically significant reductions in hypersexuality were both immediate and largely sustained, meaningful improvements in psychological inflexibility emerged more gradually and only for a minority of participants.

**Table 4:** Analysis of clinical significance.

Variable	Moment	Participants									
		P1	P2	P3	P4	P5	P6	P7	P8	P9	P10
HBI	Pre-Post	−6.52*	−2.79*	−2.79*	−11.42*	−2.56*	−5.59*	−2.3*	−9.55*	−3.96*	−4.42*
	Pre-3M-FU	−11.18*	−6.29*	−3.96*	−14.91*	−7.69*	0.46	−2.3*	−7.92*	−7.92*	−6.75*
	Pre-1Y-FU	−6.75*	−6.9*	−3.9*	−15.14*	−4.42*	0	−2.33*	−11.65*	−3.2*	−8.15*
NSS	Pre-Post	2.82*	−5*	3.45*	2.19*	−5.01*	4.39*	−0.62	0.31	0.3	0
	Pre-3M-FU	−6.27*	−3.45*	5.96*	4.7*	−3.76*	4.07*	−0.94	1.88	−1.25	2.5*
	Pre-1Y-FU	3.76*	−1.56	6.9*	5.01*	−3.13*	0.31	1.25	3.76*	0.31	1.88
AAQ-II	Pre-Post	−0.97	−0.13	0.13	−0.69	0.55	−0.97	−0.55	−1.67	−0.83	−0.41
	Pre-3M-FU	−1.39	1.95	−0.13	−1.81	0.27	−0.13	0.13	−0.9	−2.5*	−0.83
	Pre-1Y-FU	−0.69	−0.27	0	−2.64*	−0.13	0.13	−1.11	−2.5*	−0.97	−1.53
CFQ	Pre-Post	−1.79	−0.59	−0.79	−0.99	0.19	−0.99	−0.19	−1.99*	−2.9*	−0.79
	Pre-3M-FU	−0.39	3.58*	−1.19	−3.78*	−0.19	0.79	0.79	−1.39	−4.38*	−1.39
	Pre-1Y-FU	−0.99	1.19	−1.19	−4.18*	1.99*	−0.79	−1.59	−3.58*	−1.99*	−0.79
SBC BA	Pre-Post	2.1*	1.05	1.89	−0.21	0	−2.31*	1.89	−1.05	−1.05	0.63
	Pre-3M-FU	−0.42	1.89	1.05	0.63	1.68	−2.73*	2.94*	2.52*	−2.94*	2.31*
	Pre-1Y-FU	2.31*	1.26	1.26	1.89	0.21	−1.47	1.68	0.42	−2.94*	2.73*
SBC BD	Pre-Post	−1.18	0.16	0.33	1.01	−0.33	−2.53*	1.18	−2.02*	−1.35	0
	Pre-3M-FU	−1.35	0	0	−0.16	0.5	−1.85	1.85	0.67	−1.85	−0.33
	Pre-1Y-FU	−1.35	−0.16	0.16	0	0.5	−2.19*	0.67	−1.52	−1.85	0.16
MAAS	Pre-Post	−3.99*	1.33	2.51*	3.68*	0	0.28	0.75	0.73	−0.15	−1.11
	Pre-3M-FU	1.62	0.73	1.33	3.84*	1.62	−0.6	2.37*	−0.44	−0.6	0.66
	Pre-1Y-FU	−4.15*	1.02	1.33	6.21*	0.44	0.5	5.1*	3.55*	−0.6	−0.66

*Represent the Reliable Change Index (RCI). Absolute values greater than 1.96 reflect statistically reliable change at the individual level (95% confidence interval). 3M-FU, three-month follow-up; 1Y-FU, one-year follow-up; HBI, Hypersexual Behavior Inventory; NSSS, The New Sexual Satisfaction Scale-short form; AAQ, Acceptance and Action Questionnaire II; CFQ, Cognitive Fusion Questionnaire; SBC BA, Scale of Body Connection, body awareness; SBC BD, Scale of Body Connection, bodily dissociation; MAAS, Mindful Attention Awareness Scale; IMP, importance; CONS, consistency; DISCR, discrepancy.

## 4 Discussion

This study aimed to determine whether the improvements observed following the ACT-based intervention and at the three-month follow-up were sustained 1 year after the end of treatment. The within-group analysis revealed that post-treatment reductions in hypersexuality were maintained 1 year later, that reductions in craving interference observed at the three-month follow-up persisted at 1 year, and that perceived control over craving continued to increase through the one-year follow-up. Furthermore, psychological inflexibility, which had not changed immediately after the intervention, showed a reduction 1 year later. Conversely, several variables—consistency, sexual satisfaction, cognitive fusion, body awareness, bodily dissociation, and mindfulness skills—were not sensitive to the intervention. The within-subject clinical significance analysis indicated that all participants improved clinically in hypersexuality after treatment and that this improvement was maintained in 9 of 10 participants at three and 12 months, whereas only a small subset showed clinically significant change in psychological inflexibility. Overall, participants did not return to pre-treatment levels of hypersexuality 1 year after the intervention. Taken together, these findings provide preliminary evidence that the intervention was followed by durable, clinically meaningful benefits, particularly for hypersexuality, with some processes showing more gradual and limited change over time.

The maintenance of the changes in hypersexuality already observed at post-treatment and at the three-month follow-up after completing the eight-session intervention (Montesinos et al., 2024), as measured through both self-report instruments (hypersexuality) and subjective rating scales (degree of craving interference and perceived control over craving), is particularly relevant given the need for interventions that produce sustained impact in the domain of sexual health. It also addresses the scarcity of longitudinal studies examining the maintenance of treatment effects in hypersexuality. These findings contribute to the still limited body of evidence supporting the utility of ACT in addressing hypersexual behaviors (Crosby and Twohig, 2016; FirooziKhojastehfar et al., 2021; Levin et al., 2017), extending the evidence to a broader range of hypersexual manifestations beyond problematic pornography use.

It is important to note that the label used for the variable “control,” referred to perceived control over craving, assessed through a subjective scale, may appear to contradict ACT principles, which emphasize the acceptance of private events rather than efforts to regulate or eliminate them. However, the intervention did not conceptualize craving as something that participants should suppress or attempt to stop feeling. Instead, it was framed as an internal event to be noticed and observed without acting on it, while directing behavior toward actions aligned with personal values.

Otherwise, the delayed improvement in psychological inflexibility warrants particular caution. While there was a statistically significant reduction from pre-treatment to 1-year follow-up, no change was detected immediately post-treatment or at 3 months, and only two participants met criteria for clinically significant change. Alternative explanations are plausible: (a) the AAQ II is a broad, trait like measure with debated construct validity, which may limit sensitivity to change and risk conflating ACT processes with emotional distress outcomes; (b) in the absence of a control group, naturalistic improvement, regression to the mean, and context related life changes cannot be ruled out; and (c) process changes related to experiential avoidance and emotion regulation may consolidate gradually, becoming detectable only over longer intervals (Hayes et al., 2011; Strika-Bruneau et al., 2024a). In light of these constraints, any interpretation of a “late onset” change pattern (Ye et al., 2024) should be considered tentative rather than causal. Relatedly, although the AAQ II has shown solid empirical performance (Ruiz et al., 2013), methodological critiques (Strika-Bruneau et al., 2024b; Tyndall et al., 2019) and broader conceptualizations of psychological flexibility—e.g., dyadic processes involving openness to experience/deliteralization, self awareness/perspective taking, and motivation/activation (Hayes et al., 2011)—may help explain limited sensitivity in the short term. Future studies might therefore benefit from incorporating more recent process measures that address these concerns, such as the Comprehensive Assessment of Acceptance and Commitment Therapy Processes (Francis et al., 2016).

The possible interpretation that the delayed improvement in psychological inflexibility is related to clinical improvements in hypersexuality is consistent with prior evidence indicating that psychological inflexibility exerts a stronger influence on hypersexuality than related constructs such as cognitive fusion, bodily dissociation, or mindfulness skills (Ortega-Otero et al., 2023). It also aligns with findings from Rico and Montesinos (2025), whose path model identified psychological inflexibility as the strongest predictor of hypersexuality and substance use, thereby providing an empirical rationale for interventions such as ACT that target psychological inflexibility to reduce vulnerability to hypersexuality related behaviors.

Taken together—and acknowledging the small sample size—these results offer preliminary indications about potential mechanisms of change. The robust, sustained reductions in hypersexuality, along with reduced craving interference, and increased perceived control, are consistent with process focused models in which decreases in experiential avoidance and increases in values engagement play a central role in behavioral addictions and compulsive sexual behavior (e.g., Bőthe et al., 2023; Twohig et al., 2024). The emergence of longer term change in psychological inflexibility might reflect a delayed consolidation of flexibility related capacities, suggesting that early behavioral change (reductions in hypersexual responses and increases in valued action) might precede—and potentially facilitate—subsequent shifts in underlying regulatory processes.

Moreover, the high participation rate at the 1-year follow-up may reflect the acceptability and satisfaction with the ACT-based treatment, as well as its potential feasibility within the study population, which consisted primarily of gay and bisexual men and individuals living with HIV. This aligns with recent studies exploring the intersection between hypersexuality, chemsex, and mental health (Ortega-Otero, 2023; Strika-Bruneau et al., 2024a,b). Research in this area is particularly relevant given the need to implement evidence-based intervention programs aimed at improving the health of vulnerable individuals within the LGBTQIA+ community who engage in risky behaviors or problematic chemsex practices (Estudio Homosalud, 2021; Íncera-Fernández et al., 2025; Montesinos and Ortega, 2022).

Similarly, the sustained improvements in values-consistent behavior observed at follow-up may be related to the emphasis placed by ACT-based interventions on values clarification and committed action, as well as the focus on psychological acceptance rather than control or avoidance of aversive private events (Hayes et al., 2014). As previously noted (Montesinos et al., 2024), a summative rather than eliminative approach—one that prioritizes not the reduction of problematic behaviors but the cultivation of a more fulfilling life—may provide a useful foundation for addressing hypersexuality. This is particularly relevant given that functional analysis often reveals hypersexual behaviors as part of a broader pattern of actions aimed at escaping discomfort, which may be effective in the short term but ultimately becomes highly problematic and limiting.

Despite the aforementioned findings, this study presents several limitations. First, the small sample size and the absence of a control group limit the generalizability of the results and prevent causal inferences between the intervention and the observed changes. Although some of the self-report measures and subjective scales showed changes in the same direction, it is not possible to entirely rule out the influence of other factors or the mere passage of time. Therefore, given the small sample size of the present study, the findings should be interpreted with caution, and their generalizability to broader populations remains limited. In any case, and in relation to the absence of a control group, this is consistent with the exploratory nature of this pilot study, which was designed primarily to assess feasibility and preliminary signals of efficacy rather than to establish causal effects. Second, although the response rate at the 1-year follow-up was high, the loss of two participants may have affected the representativeness of the results, and it cannot be ruled out that those who did not respond experienced significantly more negative outcomes than those who did. Third, the study did not include measures of the therapeutic alliance, which could have provided valuable information regarding process variables that may account for the outcomes. Fourth, no formal or standardized measures of treatment integrity were employed. Although weekly supervision with an expert ACT clinician was used to monitor adherence by reviewing session recordings, future research should incorporate objective fidelity assessments (e.g., *ad hoc* adherence checklists or the ACT Fidelity Measure, ACT-FM; O'Neill et al., 2019). Fifth, the study did not include a systematic assessment of comorbid mental health conditions, which would have been particularly relevant given the high prevalence of psychiatric comorbidities among individuals with hypersexuality—including minority groups and chemsex users. Nevertheless, the sample did exclude participants with substance dependence (beyond sex-associated consumption), personality disorders, or chronic mental disorders. Finally, the use of self-report instruments carries the risk of biases related to social desirability, recall bias, insufficient awareness of one’s own emotional states or behaviors, acquiescence bias, respondent fatigue, ambiguous item interpretation, or halo effects. Future studies should include larger samples, experimental designs with control groups and random assignment, and should incorporate multiple assessment methods, including observational techniques or interviews alongside self-report data.

Finally, this study offers preliminary insights into the potential utility of a brief, individual ACT-based intervention for individuals experiencing hypersexuality, highlighting its promise as a feasible and sustainable therapeutic approach. The analyses have an exploratory nature, but the clinical implications of these findings are significant: they offer support for the feasibility, acceptability, and utility of a brief, process-focused intervention for a condition associated with considerable psychological distress. Moreover, these results pave the way for the design of controlled clinical trials that could confirm and expand upon these observations. In conclusion, the findings support the utility of ACT for individuals struggling with sexual impulse control, particularly among men with non-heterosexual orientations—a group that remains underrepresented in the scientific literature. If confirmed in future studies, such interventions could have a positive impact on both sexual health and overall quality of life for individuals with hypersexuality. Ultimately, and if the findings of this clinical trial are replicated, vulnerable individuals may benefit by moving closer to what they consider a fulfilling life, specifically by achieving better management of their impulses and sexual behaviors, and by relating to their distress in a more flexible and mindful way.

## Data Availability

The raw data supporting the conclusions of this article will be made available by the authors, without undue reservation.
